# Inventory rationing policy and backup supply with two classes of customers

**DOI:** 10.1371/journal.pone.0214386

**Published:** 2019-04-12

**Authors:** Mingwu Liu, Xiaomei Wei

**Affiliations:** School of Economics and Management, Chongqing Jiaotong University, Chongqing, China; Indian Institute of Technology Madras, INDIA

## Abstract

In this paper, we consider joint decisions on inventory rationing and replenishment from a regular supplier or a backup supplier with two classes of customers. When the on-hand inventory falls below a certain critical level, an inventory rationing policy is implemented to reject lower priority customers. We investigated the potential use of a backup supplier as a response to inventory rationing policy. First, we establish an inventory level transition equilibrium equation for the steady-state inventory level probability distributions and derive analytical steady-state performance indicators. Next, the inventory cost function and the nonlinear optimization model are characterized. We use real-coded genetic algorithm (RCGA) to search for the optimal joint decisions. Finally, the proposed algorithm and the joint decisions are investigated with different inventory service polices through numerical experiments. The results show that the inventory rationing policy (IRP) is superior to the traditional priority service policy (CPSP) and the common first-come-first service policy (FCFSP). The backup supply can reduce inventory costs with high arrival intensity.

## Introduction

Inventories include raw materials, work in progress and finished goods, which are important drivers in modern supply chains and have traditionally been used to alleviate uncertainty in demand and supply or to improve service levels. In real life, an inventory system that is affected by multiple classes of customers is often observed. Often, the diverse needs of multiple classes of customers lead to a variety of service priorities. The classes of priority inventory demands are usually divided into booking orders and unscheduled orders. For a distribution center, the customers who have placed a predetermined order in advance have the right to priority inventory service than those who have not placed a predetermined order. Another example is the inventory operation about a distributor ships to the downstream retailers. In the face of the product orders from the retailers with long-term contracts and the general retailers, the distributor tends to prioritize the retailers with long-term contracts. In reality, there are often situations where inventory levels are not sufficient to provide services to all customers. At this point, the inventory rationing strategy (IRP) allows for more sophisticated inventory control services. For example, the distributor can set an inventory rationing threshold in advance, and once the on-hand inventory level is below this threshold, the distributor will no longer provide inventory services to the general retailers.

It is often seen in practice that supply risk has a serious impact on the performance of inventory management. In response to uncertain supply disruption risks and mitigate the impact of supply shortage, many companies use backup procurement as the simplest but most effective procurement strategy[1]. A well-known example is the Nokia-Ericsson case in 2000, in which a fire caused Philips to close its semiconductor factory in New Mexico. Ericsson lost $400 million due to supply disruptions for several weeks, and Nokia managed to procure from alternative suppliers, minimizing harmful supply impacts[2]. GE employs two certified suppliers in different geographic locations. If the major supplier is unable to meet GE's needs on time, the alternate supplier will be used to fulfill the order[3]. Many real-world examples show that backup procurement can reduce supply risk when the regular supply process is unable to cope with unexpected events.

The above real-life examples and wide-ranging implications prompted us to consider jointly decision-making model for inventory rationing policy and backup procurement with two classes of customers. Our developed model can be used to describe the inventory management for an online store in e-commerce environment. The online store can divide the visiting customers into two classes based on the customers’ records of visits and purchase, as well as purchase reviews. Those who have a purchase record and give a praise can be marked as the priority customers, and those who give bad reviews or those who are first-time visitors are marked as the ordinary customers. These priority customers feel a pleasant buying experience. They will form a good word-of-mouth communication among the consumer groups and, therefore, have greater customer value. Such customers should be meet demands and be provided priority services when visiting the store. When the store introduces a very welcome new product, an inventory rationing strategy (IRP) can be implemented to protect the service needs of the priority customers in response to the situations in which the inventory does not meet the needs of both classes of customers. The store can replenishment from regular suppliers or backup suppliers. The backup supplier has a shorter lead time, which is in favor of reducing shortage lost. But, the backup supplier charges more of each item. The main contributions of this paper include: (1) We model an inventory rationing system with two classes customers and investigate the trade-off between inventory rationing policy and backup supply policy. (2) We formulate a queuing service model and provide an analytical procedure that accurately evaluate system performance. (3)We establish a nonlinear inventory cost model with constraints and integer variables. A real-coded genetic algorithm (RCAG) is used to solve the cost optimization problem.

The rest of the paper is organized as follows. In Section 2, we discuss relevant literature briefly. In Section 3, we give a detailed description of the inventory model assumptions. Section 4 derives steady-state performance metrics. In Section 5, we first establish a long-term average inventory cost function, and then display the cost function attributes for r, T, and Q by plotting the cost function figure profiles. An integer optimization model is also established, and a real coding genetic algorithm is proposed. Section 6 presents numerical results in specific situations. The paper will be concluded in Section 7.

## Literature review

### Inventory rationing under multiple-demand classes

In the past few decades, extensive research has been conducted on inventory rationing issues. There are two types of rationing policies: stationary and dynamic. There is a constant threshold level for the fixed inventory rationing policy. Once the inventory level drops to the threshold level, the demand from lower priority customers is no longer satisfied. Much research has been done on the stationary threshold level policy[4–5]. Isotupa introduced an inventory rationing threshold that is different from the reorder level. It demonstrates that the rationing policy can reduce inventory costs[6]. For dynamic rationing policies, threshold levels may change over time[7–8]. Fadiloğlu, et al. proposes a dynamic rationing policy for the ongoing review of the inventory system[9]. Wang and Tang studied the dynamic rationing policy in the inventory system of stock-out and sales losses[10].

Xie et al. incorporate the price reduction strategy into the inventory allocation model, and the customer is divided into two types of delivery time sensitivity[11]. The best rationing strategy is the threshold type of two customer classes. Liu at al. study the problem of inventory rationing with multiple demand classes and sales losses[12]. The optimal rationing strategy is a combination of the dynamic policy in the replenishment lead time and the static strategy before the order is placed. Bao at al. consider the dynamic inventory allocation problem for a single item with multiple demand classes and backorders[13]. The above papers discuss the classification of customers and the implementation of inventory rationing policy when the inventory is insufficient. However, another important aspect of inventory management is the inventory procurement. It is very practical to combine the inventory procurement with the inventory control.

### Supply risk and backup supply

Supply risk management can improve the performance of inventory system. Tang provides a literature review of supply risk management strategies and supply disruptions[14]. In order to mitigate the impact of supply risks on the inventory system, sourcing from a designated backup supplier is a diversified strategy [15]. Lee et al. discusses the situation in which bookstores order textbooks, in which urgent orders are designed to meet the outstanding needs of observation[16]. The cost of a rush order is usually higher than a regular order. A challenging decision is a trade-off between unsold items and the cost of unmet needs. The dual procurement strategy significantly reduces costs, but decreases as demand and production change. Zhu studied the optimal dynamic policy for inventory replenishment strategies from two sources by considering the trade-offs between different lead times, yields and costs[17]. Silbermayr and Minner analyzed the optimal allocation decisions and provide management insights on how the buyer can optimally allocate demand between the two suppliers[18]. Hou et al. study the capacity booking contract between the buyer and the alternate supplier and the disruption risk of the major supplier[1]. Different from the above papers, we focus on the jointed impact of the inventory rationing policy and the backup replenishment policy on the inventory cost performance with two classes of customers.

## The model definition

**Fig 1:**
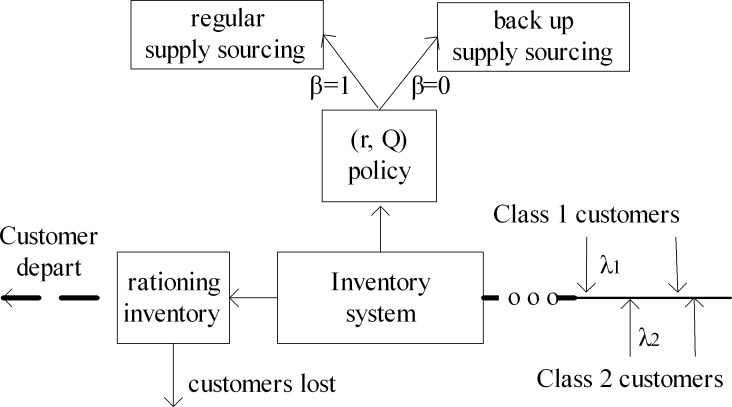
An inventory system replenishing from dual sourcing with two classes of customers.

We consider an inventory system replenishing from two different suppliers, in which there are two classes of customers (see Fig 1). Class-*i* customers arrive at the inventory system independently according to a Poisson process with rate *λ*_*i*_(*i* = 1,2) and each customer needs exactly one item from the inventory. Class-1 is considered as priority customers and Class-2 is ordinary customers. The service discipline is First-Come-First-Served (FCFS) and the service time for order processing is assumed to be 0 (Compared to the lead time for the order, the customers' order processing time can be omitted). A continuous-review (*r*,*Q*) policy is adopted, which is the most common inventory control policy. As and when the on-hand inventory drops to a prefixed reorder level *r*, an order for fixed *Q*(*Q*>*r*) units is placed. The condition *Q*>*r* ensures that there is no perpetual shortage [19]. The system will never place another order if there is an outstanding order at any time. The inventory system either replenish from a regular supplier or a backup supplier. Define a binary variable *β*, where *β* = 1 represents the order replenishing from the regular supplier and *β* = 0 shows the order replenishing from the backup supplier. It is supposed that the backup supplier will charge more for each item than the regular supplier, but the lead time for its delivery is shorter. The lead time for the order from the regular supply and the backup supply is exponentially distributed with arrival rate *μ*_1_, *μ*_2_ respectively. Exponential distribution makes the derivation to steady state equations of inventory level possible, even though it is a bad choice for modeling the replenishment lead time.

We adopt a static inventory rationing policy together with (*r*,*Q*) policy on the inventory system. We set a threshold *T*(*Q*≥*T*>0) to discriminate the two classes of customers and provide different service levels. In fact, the threshold level can be relaxed to *T*≤*r*+*Q* because the maximum possible on-hand inventory is *r*+*Q*. When the on-hand inventory is more than the threshold *T*, arrival the two classes of customers can be both served. While the on-hand inventory drops to the threshold *T*, ordinary customers who arrive are not served and hence get lost. When inventory level is zero, both classes of customers are lost. The inventory system balances the inventory out-of-stock losses and the customer churn losses through the threshold. The parameters and variables used in our model are summarized as follows:

*λ*_1_: The Poisson arrival rate of the priority customers;
*λ*_2_: The Poisson arrival rate of the ordinary customers;
*μ*_1_: The lead time parameter of exponentially for the order from the regular supplier;
*μ*_2_: The lead time parameter of exponentially for the order from the backup supplier;
*h*: the inventory holding cost per unit per unit time;
*k*: the fixed ordering cost per order;
*c*_1_: the purchase cost per unit from the regular supplier;
*c*_2_: the purchase cost per unit from the backup supplier;
*ω*_1_: the shortage cost per unit for the lost priority customers;
*ω*_2_: the shortage cost per unit for the lost ordinary customers;
*r*: The variable of inventory level for reorder;
*Q*: The variable of number of units to order;
*T*: The variable of threshold to discriminate the two classes of customers;
*β*: The binary variables of sourcing from the regular supplier or the backup supplier;
*δ*_*i*_: The 0–1 parameter for marking the Case *i*(*i* = 1,2,3).

## The steady-state performance measures

In this section, we will discuss the transition process of the on-hand inventory level in steady state and construct an equilibrium equation for the inventory level transition process. We will derive the steady-state probability distributions of the inventory level state, and calculate a series of indicators to measure the inventory system operation state. Let *I*(*t*),*t*≥0 denote the on-hand inventory level at time *t*, with state space *E* = {0,1,⋯,*T*,⋯,*r*+*Q*}. Due to Poisson arrive process of customers and exponential distribution lead-time, the next inventory level state does not depend on any past states but only on the current state. It is clearly that the inventory level process {*I*(*t*);*t*≥0} is a Markov process.

**Fig 2:**
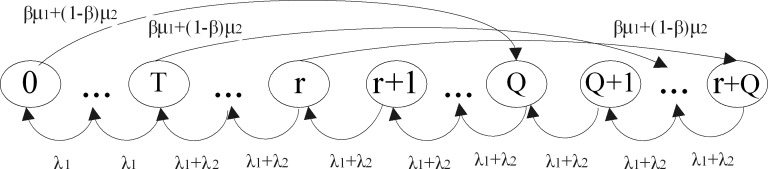
The state transition process when 0<*T*<*r*.

**Fig 3:**
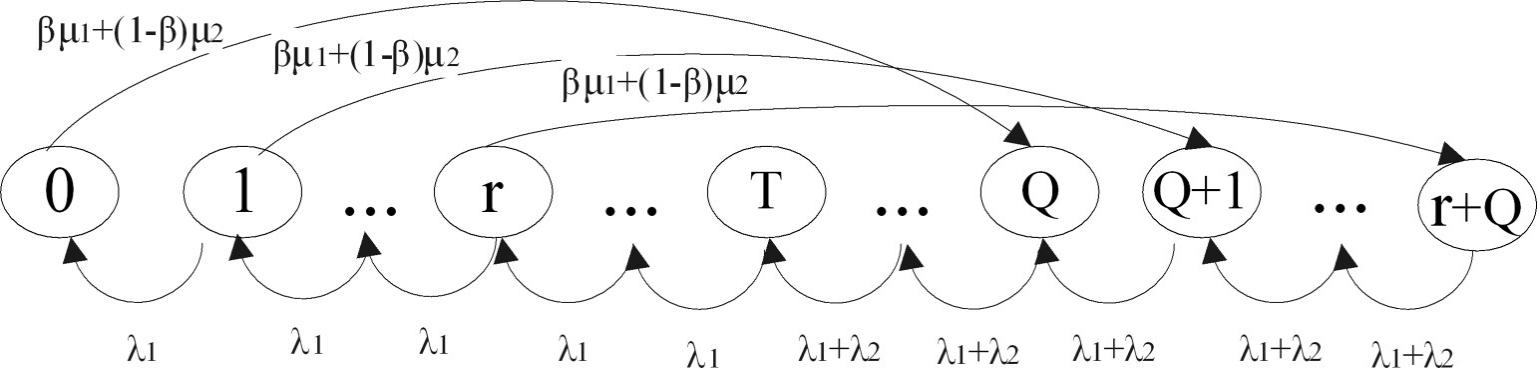
The state transition process when *r*<*T*≤*Q*.

We combine a threshold *T* and an (*r*,*Q*) order policy for the inventory system control. The size relationship between the threshold *T* and the reorder level *r* will bring about different state transition process of inventory level. If the threshold *T* is less than the reorder level *r*(0<*T*<*r*), the state transition process is depicted by Fig 2. Otherwise, the state transition process is shown by Fig 3 when *r*<*T*≤*Q*. If *T* = *r*, the figure of state transition process can be induced into Fig 2 or Fig 3.

We denote by *P*_*K*_(*i*,*j*,*t*) the state transition probability from the state *i* at time 0 to state *j* at time *t*, *P*_*K*_(*i*,*j*,*t*) = *P*_*K*_{*I*(*t*) = *j*|*I*(0) = *i*}, *i*,*j*∈*E*. *K* = 1,2,3 is used to mark Case 1 (0<*T*<*r*) and Case 2 (*r*<*T*≤*Q*) and Case 3 (*T* = *r*) respectively. We define the steady-state probability distributions of the inventory level *I*(*t*) as *P*_*K*_(*j*) = lim_*t*→∞_*P*_*K*_(*i*,*j*,*t*). When the inventory system is in a steady state, the transition-in rate is equal to the transition-out rate for each inventory level state, that is, the average number of times of entering the inventory level state per unit time is equal to the average number of times of leaving the inventory level state. The steady-state probability distributions of the inventory level will satisfy a set of state transition balance equations. The following three cases will discuss the steady inventory level state transition equations separately.

**Case 1**. The threshold level is no more than the reorder level (0<*T*<*r*).

In this situation, where *T* is less than *r*, the online store sets an threshold *T* for controlling inventory services. When the on-hand inventory level is less than *T*, the visiting ordinary customer is lost without the inventory services. When the on-hand inventory level is greater than or equal to *T*, the online store merchandise inventory is sufficient, and the priority customers and ordinary customers who visit the store can be served. According to the state transition process Fig 2, the following balance Eqs (1)–(7) can be written by the fact that transition out of a state is equal to transition into a state for a Markov process. For example, if the inventory level state *j* lines in the range *Q*≤*j*≤*Q*+*r*−1, the equation is presented in Eq (2). When *j* is within this range, there is no order pending, and then transition beyond this state can be only due to either an ordinary customer arrival or a priority customer arrival, which is presented on the left-hand side of Eq (2). Either an ordinary customer or a priority customer arrival in state *j*+1 will lead the inventory level to reduce by one unit, thus bring it to state *j*. State *j* can also be reached from state *j*−*Q* when an order with fixed *Q* arrives at intensity *Z* = *βμ*_1_+(1−*β*)*μ*_2_. The only two possible ways of reaching state *j* are reflected on the right side of Eq (2).

$$\left(\lambda_{1}+\lambda_{2}\right)P_{1}\left(r+Q\right)=\left[\beta \mu_{1}+\left(1-\beta \right)\mu_{2}\right]P_{1}\left(r\right)$$ (1)

$$\left(\lambda_{1}+\lambda_{2}\right)P_{1}\left(j\right)=\left[\beta \mu_{1}+\left(1-\beta \right)\mu_{2}\right]P_{1}\left(j-Q\right)+\left(\lambda_{1}+\lambda_{2}\right)P_{1}\left(j+1\right),\;j=Q,\cdots ,Q+r-1,$$ (2)

$$\left(\lambda_{1}+\lambda_{2}\right)P_{1}\left(j\right)=\left(\lambda_{1}+\lambda_{2}\right)P_{1}\left(j+1\right),\;j=r+1,\cdots ,Q-1,$$ (3)

$$\left[\lambda_{1}+\lambda_{2}+\beta \mu_{1}+\left(1-\beta \right)\mu_{2}\right]P_{1}\left(j\right)=\left(\lambda_{1}+\lambda_{2}\right)P_{1}\left(j+1\right),\;j=T+1,\cdots ,r,$$ (4)

$$\left[\lambda_{1}+\beta \mu_{1}+\left(1-\beta \right)\mu_{2}\right]P_{1}\left(T\right)=\left(\lambda_{1}+\lambda_{2}\right)P_{1}\left(T+1\right)$$ (5)

$$\left[\lambda_{1}+\beta \mu_{1}+\left(1-\beta \right)\mu_{2}\right]P_{1}\left(j\right)=\lambda_{1}P_{1}\left(j+1\right),\;j=1,2,\cdots ,T-1,$$ (6)

$$\left[\beta \mu_{1}+\left(1-\beta \right)\mu_{2}\right]P_{1}\left(0\right)=\lambda_{1}P_{1}\left(1\right).$$ (7)

The above set of equations with the normalizing condition ($\sum_{j=0}^{Q+r}P_{1}\left(j\right)=1$) determine the steady-state probability distributions uniquely. We solve the Eqs (1)–(7) by means of recursive process. From Eq (7), we get *P*_1_(1) = [*βμ*_1_+(1−*β*)*μ*_2_]*P*_1_(0)/*λ*_1_. Then put *P*_1_(1) into Eq (6), *P*_1_(2) will be derived. Through the similar iterative operations, we get

$$P_{1}\left(j\right)=\frac{Z}{\lambda_{1}}{\left(1+\frac{Z}{\lambda_{1}}\right)}^{j-1}P_{1}\left(0\right),\;j=1,2,\cdots ,T,$$ (8)

$$P_{1}\left(j\right)=\frac{Z}{\lambda_{1}+\lambda_{2}}(1+\frac{Z}{\lambda_{1}}{)}^{T}(1+\frac{Z}{\lambda_{1}+\lambda_{2}}{)}^{j-T-1}P_{1}\left(0\right),\;j=T+1,\cdots ,r,$$ (9)

$$P_{1}\left(j\right)=\frac{Z}{\lambda_{1}+\lambda_{2}}(1+\frac{Z}{\lambda_{1}}{)}^{T}(1+\frac{Z}{\lambda_{1}+\lambda_{2}}{)}^{r-T}P_{1}\left(0\right),j=r+1,\cdots ,Q,$$ (10)

$$P_{1}\left(j\right)=\frac{Z}{\lambda_{1}+\lambda_{2}}[(1+\frac{Z}{\lambda_{1}}{)}^{T}(1+\frac{Z}{\lambda_{1}+\lambda_{2}}{)}^{r-T}-(1+\frac{Z}{\lambda_{1}}{)}^{j-Q-1}]P_{1}\left(0\right),j=Q+1,\cdots ,Q+T,$$ (11)

$$P_{1}\left(j\right)=\frac{Z}{\lambda_{1}+\lambda_{2}}(1+\frac{Z}{\lambda_{1}}{)}^{T}[(1+\frac{Z}{\lambda_{1}+\lambda_{2}}{)}^{r-T}-(1+\frac{Z}{\lambda_{1}+\lambda_{2}}{)}^{j-Q-T-1}]P_{1}\left(0\right),\;j=Q+T+1,\cdots ,Q+r,$$ (12)

$$P_{1}\left(0\right)=(\lambda_{1}+\lambda_{2})[QZ(1+\frac{Z}{\lambda_{1}}{)}^{T}(1+\frac{Z}{\lambda_{1}+\lambda_{2}}{)}^{r-T}+\lambda_{2}(1+\frac{Z}{\lambda_{1}}{)}^{T}+\lambda_{1}{]}^{-1}.$$ (13)

Inserting (13) into (8)-(12) respectively, we have the analytical steady-state probability distributions of the inventory level for Case 1.

**Case 2**. The threshold level is higher than the reorder level (*r*<*T*≤*Q*).

In this case, the threshold *T* is larger relative to CASE1. The online store provides more stringent service conditions for the ordinary customers. The ordinary customers can get inventory services through the online store only if the on-hand inventory level is greater than or equal to *T*. Similar to Case 1, we have the following analytical probability distributions of the inventory level for Case 2.

$$P_{2}\left(j\right)=\frac{Z}{\lambda_{1}}(1+\frac{Z}{\lambda_{1}}{)}^{j-1}P_{2}\left(0\right),\;j=1,2,\cdots ,r,$$ (14)

$$P_{2}\left(j\right)=\frac{Z}{\lambda_{1}}(1+\frac{Z}{\lambda_{1}}{)}^{r}P_{2}\left(0\right),\;j=r+1,\cdots ,T,$$ (15)

$$P_{2}\left(j\right)=\frac{Z}{\lambda_{1}+\lambda_{2}}(1+\frac{Z}{\lambda_{1}}{)}^{r}P_{2}\left(0\right),\;j=T+1,\cdots ,Q,$$ (16)

$$P_{2}\left(j\right)=\frac{Z}{\lambda_{1}+\lambda_{2}}[(1+\frac{Z}{\lambda_{1}}{)}^{r}-(1+\frac{Z}{\lambda_{1}}{)}^{j-Q-1}]P_{2}\left(0\right),\;j=Q+1,\cdots ,Q+r,$$ (17)

$$P_{2}\left(0\right)=(\lambda_{1}+\lambda_{2})[\lambda_{1}+(\lambda_{2}+\frac{\lambda_{2}Z(T-r)}{\lambda_{1}}+ZQ)(1+\frac{Z}{\lambda_{1}}{)}^{r}{]}^{-1}.$$ (18)

**Case 3.** The threshold level is same as the reorder level (*T* = *r*).

In this case, the inventory rationing policy proposed in this paper degenerates into a common (*r*,*Q*) priority policy, which is the same as the inventory service policy in [20]. The steady-state probability distributions *P*_3_(*j*),*j* = 1,⋯,*r*+*Q* are given by

$$P_{3}\left(j\right)=\frac{Z}{\lambda_{1}}(1+\frac{Z}{\lambda_{1}}{)}^{j-1}P_{3}\left(0\right),\;j=1,2,\cdots ,r$$ (19)

$$P_{3}\left(j\right)=\frac{Z}{\lambda_{1}+\lambda_{2}}(1+\frac{Z}{\lambda_{1}}{)}^{r}P_{3}\left(0\right),j=r+1,\cdots ,Q,$$ (20)

$$P_{3}\left(j\right)=\frac{Z}{\lambda_{1}+\lambda_{2}}[(1+\frac{Z}{\lambda_{1}}{)}^{r}-(1+\frac{Z}{\lambda_{1}}{)}^{j-Q-1}]P_{3}\left(0\right),\;j=Q+1,\cdots ,Q+r,$$ (21)

$$P_{3}\left(0\right)=(\lambda_{1}+\lambda_{2})[QZ(1+\frac{Z}{\lambda_{1}}{)}^{r}+\lambda_{2}(1+\frac{Z}{\lambda_{1}}{)}^{r}+\lambda_{1}{]}^{-1}.$$ (22)

Before setting up an inventory cost model, we firstly calculate several important steady-state performance measures, which are used to for future inventory system control. In this inventory system, the optimums of *T* and *r* should be determined simultaneously. We do not know the relationship between *T* and *r* in advance. For the convenience of building a optimizing model including Case 1, Case 2 and Case 3, we denote three 0–1 parameters as $\delta_{1}=\left\{ \begin{array}{l} 1,\;0<T<r\\ 0,\;otherwise \end{array}\right.$, $\delta_{2}=\left\{ \begin{array}{l} 1,\;r<T\leq Q\\ 0,\;otherwise \end{array}\right.$ and $\delta_{3}=\left\{ \begin{array}{l} 1,\;r=T\\ 0,\;otherwise \end{array}\right.$ to mark the three cases separately. Let ${\bar{I}}_{n}$ denote the average inventory. We have

$${\bar{I}}_{n}=\delta_{1}\sum_{j=1}^{r+Q}jP_{1}\left(j\right)+\delta_{2}\sum_{j=1}^{r+Q}jP_{2}\left(j\right)+\delta_{3}\sum_{j=1}^{r+Q}jP_{3}\left(j\right)$$ (23)

Denote the mean reorder rate, the mean shortage rates for the priority and ordinary customer as ${\bar{R}}_{re}$, ${\bar{\psi }}_{pri}$ and ${\bar{\psi }}_{ord}$ respectively. According to the (*r*,*Q*) policy, the inventory level reaching the reorder level *r* will trigger an order at once. For Case 1, ether the priority customer or the ordinary customer arrival can trigger an order when the on-hand inventory level is *r*+1(>*T*). For Case 2, a reorder is triggered only duo to a demand from the priority customer when the on-hand inventory level is *r*+1(≤*T*). Because a demand from an ordinary customer will be rejected when the on-hand inventory level is *r*+1(≤*T*). For case 3, there is only one threshold *T*(*T* = *r*). Each customer arrival can trigger an order when the on-hand inventory level is *r*+1. The mean reorder rate is

$${\bar{R}}_{re}=\delta_{1}\left(\lambda_{1}+\lambda_{2}\right)P_{1}\left(r+1\right)+\delta_{2}\lambda_{1}P_{2}\left(r+1\right)+\delta_{3}\left(\lambda_{1}+\lambda_{2}\right)P_{3}\left(r+1\right)$$ (24)

A demand coming from the priority customers causes a shortage when the on-hand inventory level is 0 for Case1, Case 2 and Case 3. An ordinary customer is rejected service and the demand will be lost when the on-hand inventory level is no more than the rationing threshold *T* in Case1, Case 2 and Case 3. Hence, the shortage rate for the priority customer and the ordinary customer are given by (25) and (26) respectively.

$${\bar{\psi }}_{pri}=\delta_{1}\lambda_{1}P_{1}\left(0\right)+\delta_{2}\lambda_{1}P_{2}\left(0\right)+\delta_{3}\lambda_{1}P_{3}\left(0\right),$$ (25)

$${\bar{\psi }}_{ord}=\delta_{1}\lambda_{2}\sum_{j=0}^{T}P_{1}\left(j\right)+\delta_{2}\lambda_{2}\sum_{j=0}^{T}P_{2}\left(j\right)+\delta_{3}\lambda_{2}\sum_{j=0}^{T}P_{3}\left(j\right).$$ (26)

## The optimization model and solution algorithm

### Cost analysis

In this section, we establish a steady-state average inventory cost function per unite time. The system incurs inventory holding costs, fixed ordering costs, purchase costs and costs of lost for possibly rejecting two classes of customers’ order. Using the definitions of each cost component numbered above, the total long-run average inventory cost function per unit time is given by

$$Cost\left(r,T,Q,\beta \right)=h{\bar{I}}_{n}+k{\bar{R}}_{re}+\beta c_{1}Q{\bar{R}}_{re}+(1-\beta )c_{2}Q{\bar{R}}_{re}+\omega_{1}{\bar{\psi }}_{pri}+\omega_{2}{\bar{\psi }}_{ord}$$ (27)

Where, *r*, *T*, *Q* are integer variables and *β* is a binary variable with 0 or 1. The first item $h\bar{I}$ is the inventory holding cost. The $k{\bar{R}}_{re}$ is the fixed ordering cost. The $\beta c_{1}Q{\bar{R}}_{re}$ is the inventory purchasing cost from the regular supplier. The $(1-\beta )c_{2}Q{\bar{R}}_{re}$ is the inventory purchasing cost from the back supplier. And $\omega_{1}{\bar{\psi }}_{pri}$ and $\omega_{2}{\bar{\psi }}_{ord}$ are the costs of lost for the priority customer and the ordinary customers respectively.

**Fig 4:**
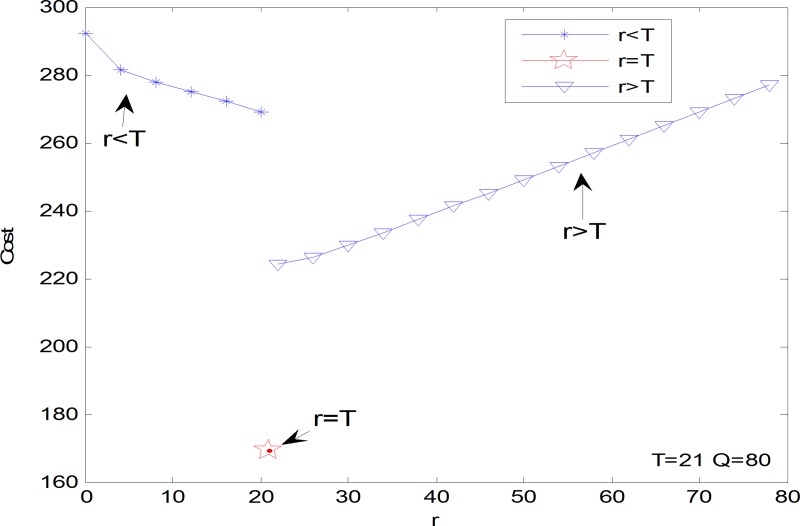
The inventory cost as a function of *r*.

**Fig 5:**
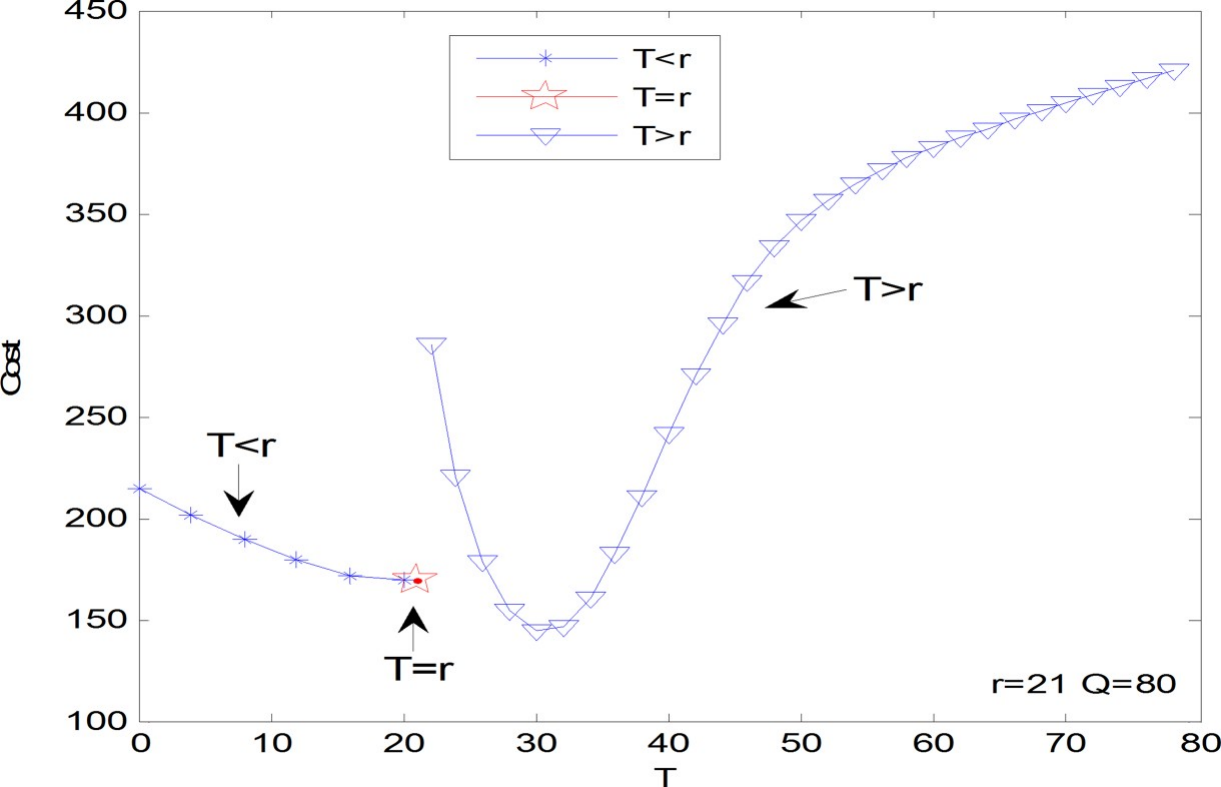
The inventory cost as a function of *T*.

**Fig 6:**
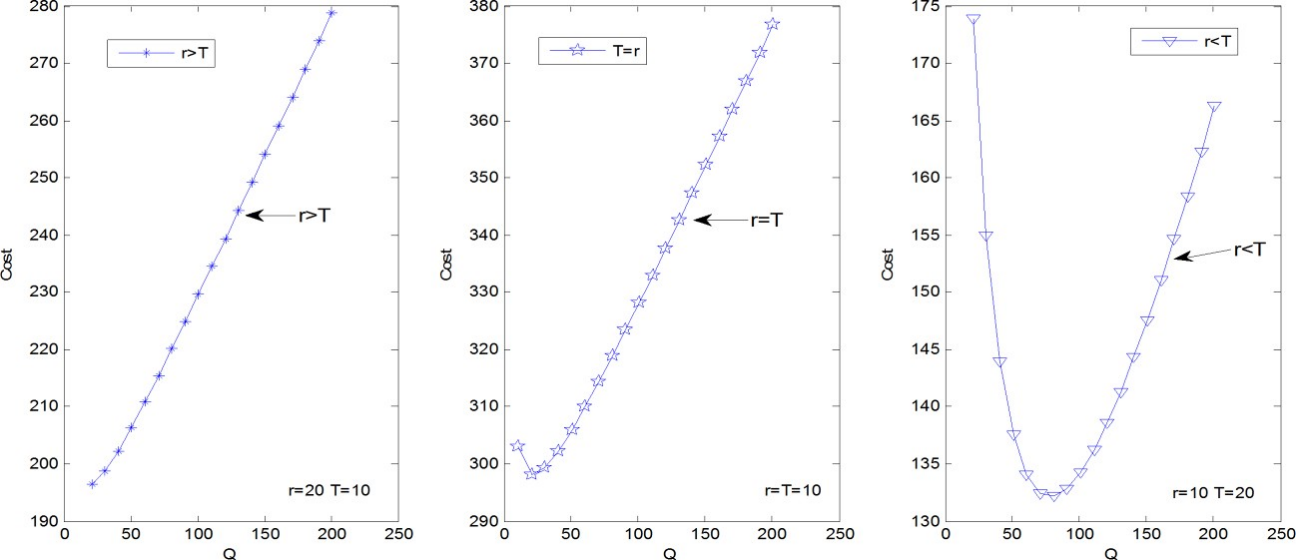
The inventory cost as a function of *Q*.

It is hard to analytically figure out any good property of the inventory cost function. The numerical investigation can indicate properties of the cost function of *r*, *T* and *Q* respectively. Let *λ*_1_ = 10, *λ*_2_ = 10, *μ*_1_ = 5, *μ*_2_ = 10, *ω*_1_ = 60, *ω*_2_ = 20, *c*_1_ = 10, *c*_2_ = 15, *h* = 1, *k* = 10. Figs 4–6 shows the inventory cost has a unique minimum as a function of *r*, *T* and *Q* respectively. Note that the property of parameter *β* need not to be obtained because *β* is a binary variable. Figs 4–6 shows how the actual online store can set the optimal inventory control decision variables one by one. Take the decision of the threshold *T* for example, the online store first checks the inventory cost at *T* = *r*, and then gradually increases the *T* value until the corresponding inventory cost shifts from a gradual decline to a gradual increase. The *T* value of this turning point is the optimal inventory control threshold. This kind of practice can only be optimized for a single variable, and the joint optimal solution of multiple variables cannot be obtained. It must be obtained with the help of modern intelligent algorithms such as the genetic algorithm.

### Optimization model

Here, an integer optimization model is setup to obtain the optimal joint decision on the inventory rationing and the backup supply. The inventory system minimizes the total long-term average inventory cost function per unit time as the decision goal, and uses *r*,*T*,*Q*,*β* as the decision variables to make the optimal joint decision. The optimization model is shown below:

$$MinCost(r,T,Q,\beta )=Min_{\left(r,T,Q,\beta \right)}\left\{h{\bar{I}}_{n}+k{\bar{R}}_{re}+\beta c_{1}Q{\bar{R}}_{re}+(1-\beta )c_{2}Q{\bar{R}}_{re}+\omega_{1}{\bar{\psi }}_{pri}+\omega_{2}{\bar{\psi }}_{ord}\right\},$$ (28)

Subject to:

r−Q≤−1, (29)

0<T≤Q, (30)

0≤r, (31)

The objective function minimizes the inventory cost represented by (28). Constraint (29) assumes that there is no permanent shortage. Constraint (30) limits the rationing action space to no more than the order quantity level. Constraint (31) indicates that the reorder point is greater than 0.

*β* is a binary variable with 0 or 1, *r*, *T* and *Q* are integer variables.

### Algorithm designing

Due to the complexity of the inventory cost function, it is not been possible to derive an explicit expression for the optimal inventory control policy. As constraint (29) is linear, the optimization can be based on the method by enumerating and bounding but with large mounts of calculations. Several classical computational techniques (such as branch and bound technique, cutting planes technique, et al.), which are reasonably efficient, can be applicable to a class of problems. But, in the case of non-convex problems these techniques may cut-off the global optima [21]. Some evolutionary computational techniques (such as genetic algorithms, GA; Particle Swarm optimization, PSO, etc.) have been successfully applied in the field of integer optimization; and, they get better results calculation methods in a faster and cheaper way than non-evolution computation methods. For example, the genetic algorithms is a method for solving constrained and unconstrained optimization problems. It can be applied to solve various optimization problems that are not suitable for standard optimization algorithms, including discontinuous, non-differentiable, random or highly nonlinear problems.

This paper adopts a real coded genetic algorithm (RCGA) introduced by [21] with an efficient constraint handling method proposed by [22]for solving integer non-linear optimization problems with constraints. RCGA, which uses a real number representation, has fast convergence towards optima than binary and gray coded GA and can overcome the difficulty of Hamming Cliff as in binary coded GA. Parameter free, penalty function approach based on feasibility approach introduced by [22] is used in the constraint handling. A parameter-less penalty function method based on the feasibility method introduced by [22] is used for constraint processing. The algorithm procedures are summarized as follows:

Step 1. Generate a suitably large initial set of populations within the domain specified by the variable boundary, i.e. a random point to satisfy 0≤*β*≤1, *r*≥0, *r*+1≤*Q* and *T*<*Q*.

Step 2. Check the stopping standard for the algorithm. Stop, if the standard is reached, otherwise go to Step 3.

Step 3. A tournament selection program is applied on the initial population to create a mating pool.

Step 4. Laplace-crossover and Power-mutations are applied to all individuals in the mating pool to generate an new population. Apply integer restrictions on decision variables.

Step 5. Evaluate their fitness values and go to step 2. Otherwise, increase generation, go to Step 3.

The algorithm can be put in practice by Optimization Tool on MATLAB R2013a. Maiti et al. pointed out that there is no uniform standard for population size setting in genetic algorithms[23]. If the population is too large, there gives rise to some difficulties in storing of the data. But if the population size is too small, there may not be enough populations for good crossover[24–25]. We determine the population size by several attempts.

## Numerical analysis

Here, we present numerical analysis for comparisons. First, we compare the performance of the improved genetic algorithm with the optimal solutions obtained by complete enumeration. Secondly, we compare with several commonly used inventory service policies in order to identify the conditions in which rationing and backup supply is important. Finally, we investigate the sensitivities of relative arrival rate of order (*μ*_2_/*μ*_1_), relative shortage cost (*ω*_2_/*ω*_1_) and relative order price (*c*_2_/*c*_1_) and find out in what situations backup supply can reduce inventory cost and offset the influence of inventory rationing.

(1) We consider an inventory system with the parameters as *λ*_1_ = 10, *λ*_2_ = 10, *μ*_1_ = 5, *μ*_2_ = 10, *ω*_1_ = 60, *ω*_2_ = 20, *c*_1_ = 10, *c*_2_ = 15, *k* = 100, *h* = 1. The optimal inventory control policy is computed with the use of the improved genetic algorithm presented in Section 5. The algorithm can be put in practice with the initial population of 100 individuals, the crossover probability Pc = 0.85, and mutation probability Pm = 0.1. The algorithm stops when the number of generations reaches the value of Generations (100) or the weighted average change of the fitness function value is less than Function tolerance (1e-6). The optimal solution(r*,T*,Q*,β*) is (8, 5, 60, 1). The corresponding steady-state performance measures are Cost* = 226.05, ${\bar{I}}_{n}=35.81$, ${\bar{R}}_{re}=0.2616$, ${\bar{\psi }}_{pri}=0.0441$ and ${\bar{\psi }}_{ord}=0.2232$. We find that the improved genetic algorithm provides the same solutions as the complete enumeration.

(2) We compare inventory rationing policy (IRP), classical priority service policy (CPSP) and ordinary First-Come-First-Served policy (FCFSP). The IRP is the control policy of customers classification presented in this paper, which has a threshold *T*. The CPSP is the same as the policy in [20], in which the threshold *T* is equal to the reorder level *r*. The FCFSP do not discriminate any arrival customer class, which is same as the ordinary (*r*, *Q*) policy. Let us consider the parameter *λ*_1_ and *λ*_2_ in a situation where *μ*_1_ = 5, *μ*_2_ = 10, *ω*_1_ = 60, *ω*_2_ = 20, *c*_1_ = 10, *c*_2_ = 15, *k* = 100, *h* = 1.

**Fig 7:**
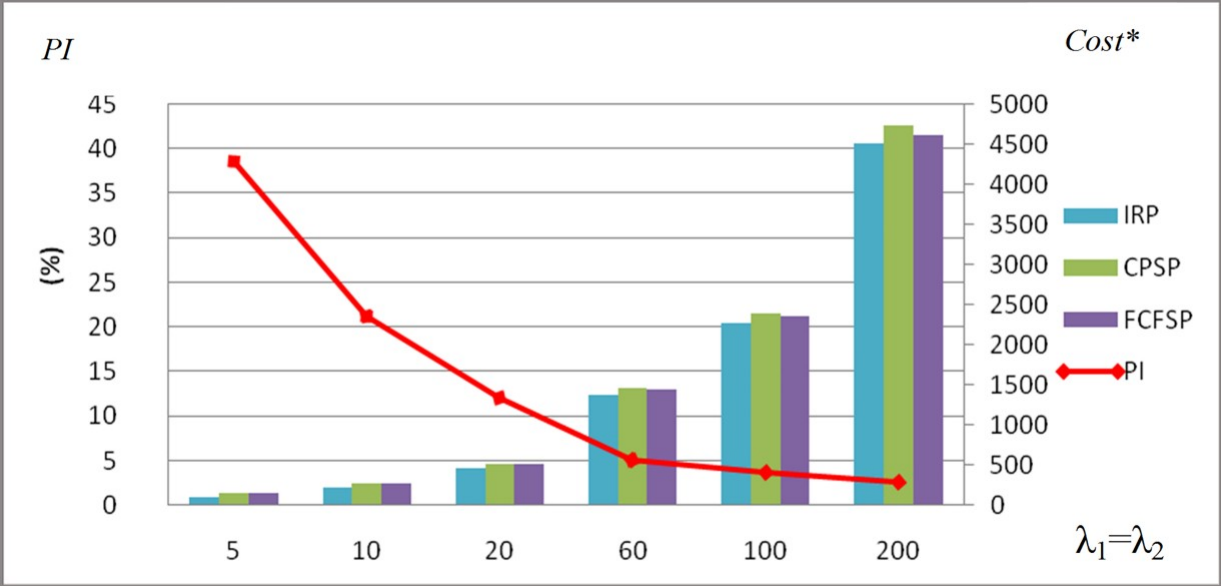
The performance of the inventory rationing policy.

The results are reported in Table 1 and Table 2. We define a performance measure index $PI=\frac{Min(Cost_{CPSP},Cost_{FCFSP})-Cost_{IRP}}{Cost_{IRP}}\times 100\%$. This index represents the performance of the IRP compared to the CPSP and the FCFSP and the results are presented in Fig 7. We find that the IRP is superior to the CPSP and the FCFSP at the same arrival intensity or different arrival intensity of the two classes of customers. For example, the performance measure index *PI* is equal to 37.6% at *λ*_1_ = *λ*_2_ = 5, which indicates the IRP is significantly better than the CPSP and the FCFSP. Fig 7 shows that as the arrival intensity increases, the *PI* decreases. The *PI* is equal to 2.55% at *λ*_1_ = *λ*_2_ = 200. The IRP perform worse in the situation of higher customer arrival intensity because of rejecting more ordinary customers. The CPSP is superior to the FCFSP at low customer arrival intensity, while is inferior to the FCFSP at high arrival intensity. For example, the optimum cost under CPSP and FCFSP is 149.71 and 150.77 at *λ*_1_ = *λ*_2_ = 5. While the optimum cost under CPSP and FCFSP is 2395.68 and 2353.41 at *λ*_1_ = *λ*_2_ = 100. The CPSP, in which the reorder level *r* is considered as the inventory rationing threshold, will lost some flexibility in the situation of high customer arrival intensity compared to the FCFSP. So it is necessary to introduce a rationing threshold *T* and the threshold is only used as rejecting or accepting an arrival customer. This paper is also motivated by the interesting findings. The optimum of reorder level (r*) is always higher than the optimal rationing threshold (T*). The inventory system always orders from the regular supplier (β* = 1), because the difference of the lead time of the two suppliers is small. We anticipate that the lead time will have an impact on the decision-making of placing an order.

**Table 1:** Comparison of different polices at the same arrival intensity the two classes of customers.

*λ*_1_ = *λ*_2_	IRP	CPSP	FCFSP
	(r*,T*,Q*,β*), Cost*	(r*,Q*,β*), Cost*	(r*,Q*,β*), Cost*
5	(4, 3, 37, 1), 108.08	(3, 47, 1), 149.71	(4, 47, 1), 150.77
10	(8, 5, 60, 1), 226.05	(6, 70, 1), 274.28	(9, 68, 1), 275.59
20	(18, 8, 91, 1), 459.8	(12, 107, 1), 515.4	(21, 97, 1), 515.45
60	(65, 21, 175, 1), 1372.22	(40, 230, 1), 1458.94	(72, 180, 1),1442.12
100	(116, 34, 239, 1), 2270.64	(67, 341, 1), 2395.68	(129, 242, 1), 2353.41
200	(254, 67, 367, 1), 4497.43	(137, 634, 1), 4732.57	(278, 371, 1), 4612.03

The * in the table represents the optimal solution.

**Table 2:** Comparison of different polices at the different arrival intensity of the two classes of customers.

*λ*_2_(*λ*_1_ = 25)	IRP	CPSP	FCFSP
	(r*,T*,Q*,β*), Cost*	(r*,Q*,β*), Cost*	(r*,Q*,β*), Cost*
5	(23, 10, 30, 1), 305.53	(15, 86, 1), 396.49	(17, 84, 1), 396.90
10	(26, 10, 32, 1), 359.31	(15, 95, 1), 456.13	(20, 91, 1), 456.57
20	(30, 10, 39, 1), 467.02	(16, 113, 1), 575.08	(24, 104, 1), 574.64
30	(35, 10, 44, 1), 574.52	(16, 132, 1), 693.76	(29, 116, 1), 691.51
40	(40, 10, 50, 1), 682.65	(16, 151, 1), 812.28	(34, 127, 1), 807.5

The * in the table represents the optimal solution.

(3) Comparison the optimal IRP in a situation with low, middle and high arrival intensity. The experiment will reveal the insensitivity of lead time. Let us consider the parameter *μ*_2_ a situation where *μ*_1_ = 5, *ω*_1_ = 60, *ω*_2_ = 20, *c*_1_ = 10, *c*_2_ = 15, *k* = 100, *h* = 1. The results are presented in Table 3. In the situation with low arrival intensity (*λ*_1_ = *λ*_2_ = 10), the system order from the regular supplier when *μ*_2_≤10. As the *μ*_2_ increases, the system will turn to order from the backup supplier. The system orders from the backup supplier when the *μ*_2_ reaches 50 in a situation of *λ*_1_ = *λ*_2_ = 50 or the *μ*_2_ reaches 90 in a situation of *λ*_1_ = *λ*_2_ = 100. The higher arrival intensity (*λ*_1_ = *λ*_2_), the system turns to order from the backup supplier at higher *μ*_2_ for the backup supply offsetting influences of inventory rationing. The results also show that the Cost* decreases as the *μ*_2_ increases when the system order from the backup supplier. The reorder level is always higher than the rationing threshold.

**Table 3:** Comparison of the lead time of the regular supplier and the backup supplier at different arrival intensity.

*μ*_2_(*μ*_1_ = 5)	*λ*_1_ = *λ*_2_ = 10	*λ*_1_ = *λ*_2_ = 50	*λ*_1_ = *λ*_2_ = 100
	(r*,T*,Q*,β*), Cost*	(r*,T*,Q*,β*), Cost*	(r*,T*,Q*,β*), Cost*
5	(8, 5, 60, 1), 226.05	(53, 18, 157, 1), 1146.09	(116, 34, 239, 1), 2270.64
10	(8, 5, 60, 1), 226.05	(53, 18, 157, 1), 1146.09	(116, 34, 239, 1), 2270.64
20	(3, 2, 45, 0), 198.23	(53, 18, 157, 1), 1146.09	(116, 34, 239, 1), 2270.64
30	(2, 1, 41, 0), 163.01	(53, 18, 157, 1), 1146.09	(116, 34, 239, 1), 2270.64
40	(2, 1, 37, 0), 139.25	(53, 18, 157, 1), 1146.09	(116, 34, 239, 1), 2270.64
50	(2, 1, 34, 0), 122.12	(5, 4, 117, 0), 1121.14	(116, 34, 239, 1), 2270.64
60	(2, 1, 32, 0), 109.16	(5, 4, 112, 0), 1054.61	(116, 34, 239, 1), 2270.64
90	(2, 1, 27, 0), 83.96	(4, 3, 103, 0), 898.423	(6, 5, 167, 0), 2242.01

The * in the table represents the optimal solution.

In fact, the order decision making is determined by relative size of system parameters of the two classes of customers, which mainly include relative arrival rate of order (*μ*_2_/*μ*_1_), relative shortage cost (*ω*_2_/*ω*_1_) and relative order price (*c*_2_/*c*_1_).

Compare the optimal IRP in three situations with low relative shortage cost, middle relative shortage cost and relative shortage cost respectively. We consider the parameter *μ*_2_ a situation where *μ*_1_ = 5, *c*_1_ = 10, *c*_2_ = 15, *k* = 100, *h* = 1. Table 4 summarizes the results. The system orders from the regular supplier in the three situations at *μ*_2_ = 10. While the system orders for the backup supplier in the three situations when *μ*_2_≥10.

**Table 4:** Comparison of the relative arrival rate of order and the relative shortage cost.

*μ*_2_(*μ*_1_ = 5)	*ω*_1_ = 60,*ω*_2_ = 20	*ω*_1_ = 100,*ω*_2_ = 20	*ω*_1_ = 1000,*ω*_2_ = 20
	(r*,T*,Q*,β*), Cost*	(r*,T*,Q*,β*), Cost*	(r*,T*,Q*,β*), Cost*
10	(8, 5, 60, 1), 226.05	(9, 6, 60, 1), 227.47	(15, 12, 60, 1), 233.30
20	(3, 2, 45, 0), 198.23	(3, 2, 45, 0), 198.72	(5, 4, 46, 0), 200.91
30	(2, 1, 41, 0), 163.01	(3, 2, 40, 0), 163.50	(4, 3, 41, 0), 165.12
40	(2, 1, 37, 0), 139.25	(2, 1, 37, 0), 139.61	(4, 3, 37, 0), 141.07

The * in the table represents the optimal solution.

Compare the optimal IRP in three situations with low relative order price, middle relative order price and high relative order price respectively. We consider the parameter *μ*_2_ a situation where *μ*_1_ = 5, *ω*_1_ = 60, *ω*_2_ = 20, *k* = 100, *h* = 1. Table 5 summarizes the results. The system orders from the backup supplier at *c*_2_ = 15. The system orders for the backup supplier at *c*_2_ = 30, *μ*_2_≥50 or at *c*_2_ = 50, *μ*_2_≥90. The higher the relative order price, the system turns to purchase from the backup supplier at higher relative arrival rate of order.

**Table 5:** Comparison of the relative arrival rate of order and the relative order price.

*μ*_2_(*μ*_1_ = 5)	*c*_2_ = 15,*c*_1_ = 10	*c*_2_ = 30,*c*_1_ = 10	*c*_2_ = 50,*c*_1_ = 10
	(r*,T*,Q*,β*), Cost*	(r*,T*,Q*,β*), Cost*	(r*,T*,Q*,β*), Cost*
20	(3, 2, 45, 0), 198.23	(8, 5, 60, 1), 226.05	(8, 5, 60, 1), 226.05
30	(2, 1, 41, 0), 163.01	(8, 5, 60, 1), 226.05	(8, 5, 60, 1),226.05
40	(2, 1, 37, 0), 139.25	(8, 5, 60, 1), 226.05	(8, 5, 60, 1), 226.05
50	(2, 1, 34, 0), 122.12	(2, 1, 34, 0), 207.67	(8, 5, 60, 1), 226.05
60	(2, 1, 32, 0), 109.16	(2, 4, 32, 0), 184.05	(8, 5, 60, 1), 226.05
90	(2, 1, 27, 0), 83.96	(2, 1, 27, 0), 138.46	(2, 1, 27, 0), 211.13

The * in the table represents the optimal solution.

## Conclusions and extensions

Motivated by a study of inventory rationing and supply-side tactics, we consider an inventory control policy supporting two classes of customers and sourcing from two suppliers. More specifically, we develop an inventory model for selecting policy parameters and analyzing performance of a rationing inventory policy and an ordering policy under a queueing inventory framework. Our model includes several practical features such as backup supply, two classes of customers, integer decision-making variables, which have not been considered simultaneously in the literatures. By using queueing analysis techniques, we derive an explicit form of the steady-state probability distribution of the inventory levels. Several inventory system performance metrics are obtained for inventory control, including average inventory, reordering rate, shortage cost, and average inventory cost. Some properties of the average inventory cost are revealed by numerical methods, which helps to design an efficient algorithm for finding the optimal solution. We built an integer optimization model for inventory control and used an effective RCGA to get the best solution. Numerical results indicate that the inventory rationing policy outperforms other commonly used inventory control policies and the backup supply can reduce inventory cost at higher relative arrival rate of order for backup supplier. The paper contributes to the literature by integrating decisions related to customer classification, backup supply and inventory optimization under an integrated queueing framework, which is different from the EOQ model, the newsvendor model and other stochastic inventory models. The model we presented here can be applied to better manage retail inventory.

In reality, the developed technique should be weighted carefully. The implementation of the IPR proposed in our paper requires more investment than these traditional polices, such as adding employees to identify ordinary customers and priority customers. It will generate inventory management costs. This question really deserves further study.
